# Classification of Gliomas and Germinomas of the Basal Ganglia by Transfer Learning

**DOI:** 10.3389/fonc.2022.844197

**Published:** 2022-03-03

**Authors:** Ningrong Ye, Qi Yang, Ziyan Chen, Chubei Teng, Peikun Liu, Xi Liu, Yi Xiong, Xuelei Lin, Shouwei Li, Xuejun Li

**Affiliations:** ^1^ Department of Neurosurgery, Xiangya Hospital, Central South University, Changsha, China; ^2^ Hunan International Scientific and Technological Cooperation Base of Brain Tumor Research, Xiangya Hospital, Central South University, Changsha, China; ^3^ Department of Neurosurgery, Sanbo Brain Hospital, Capital Medical University, Beijing, China

**Keywords:** germinoma, glioma, deep neural network, machine learning, transfer learning

## Abstract

**Background:**

Germ cell tumors (GCTs) are neoplasms derived from reproductive cells, mostly occurring in children and adolescents at 10 to 19 years of age. Intracranial GCTs are classified histologically into germinomas and non-germinomatous germ cell tumors. Germinomas of the basal ganglia are difficult to distinguish based on symptoms or routine MRI images from gliomas, even for experienced neurosurgeons or radiologists. Meanwhile, intracranial germinoma has a lower incidence rate than glioma in children and adults. Therefore, we established a model based on pre-trained ResNet18 with transfer learning to better identify germinomas of the basal ganglia.

**Methods:**

This retrospective study enrolled 73 patients diagnosed with germinoma or glioma of the basal ganglia. Brain lesions were manually segmented based on both T1C and T2 FLAIR sequences. The T1C sequence was used to build the tumor classification model. A 2D convolutional architecture and transfer learning were implemented. ResNet18 from ImageNet was retrained on the MRI images of our cohort. Class activation mapping was applied for the model visualization.

**Results:**

The model was trained using five-fold cross-validation, achieving a mean AUC of 0.88. By analyzing the class activation map, we found that the model’s attention was focused on the peri-tumoral edema region of gliomas and tumor bulk for germinomas, indicating that differences in these regions may help discriminate these tumors.

**Conclusions:**

This study showed that the T1C-based transfer learning model could accurately distinguish germinomas from gliomas of the basal ganglia preoperatively.

## Introduction

Germ cell tumors (GCTs) are neoplasms derived from reproductive cells, mostly occurring in children and adolescents at 10 to 19 years of age (1). Intracranial GCTs are classified histologically into germinomas (assessed in this study) and non-germinomatous germ cell tumors. Intracranial germinomas mostly arise from pineal or suprasellar regions (2, 3). Due to the adjacency to midbrain structures, intracranial germinoma patients usually develop hydrocephalus-related symptoms (3), and germinomas of the basal ganglia are difficult to distinguish from gliomas, which are the most common intracranial solid tumors, at the same site, even for experienced neurosurgeons or radiologists. Intracranial germinoma has a lower incidence rate compared with glioma in children and adults (4).

Intracranial germinoma is sensitive to radiation therapy, and a satisfactory prognosis could be achieved without surgical operation; however, as mentioned above, it is difficult to diagnose by routine MRI (T1-weighted, T2-weighted and enhanced T1) without additional acquisition like DWI and SWI. Unfortunately, such images are not always available due to the patient’s financial status or scanner machine-hour shortage in developing countries. Most entry-level hospitals in China are not equipped with an advanced 3T MRI scanner or haven’t purchased those additional imaging modalities from scanner vendors. *In situ* biopsies obtained intraoperatively or preoperatively with the stereotactic guide represent the “gold standard” to diagnose germinomas for most of the cases (3, 5), while the potential risk of tumor seeding or spread cannot be ignored. For some patients, traumatic procedures like surgery can be avoided if it is possible to accurately distinguish germinomas from gliomas with routine MRI. Although germinoma is commonly seen in adolescents while glioma has a higher incidence in the elderly population, headache and dizziness are common symptoms reported by both glioma and germinoma patients. Similarities in clinical manifestations compared to glioma in addition to the rarity of germinoma cases make it difficult to distinguish these two types of tumor, despite the difference of age at diagnosis. The lower incidence rate of germinoma and the abovementioned similarities provide little motivations for physicians to require additional serum test in clinics for β-HCG, an important indicator of germinoma. Therefore, a model using only routine MRI that could help physicians decide whether a patient needs further lab examination before hospital admission in such a scenario would be valuable; furthermore, a simple system requiring minimal input information that could distinguish these two types of tumor would reduce the cost per patient by cutting down unnecessary tests.

Recent advances in artificial intelligence (AI) in the field of tumor medical imaging have revealed that computers can achieve better accuracy in classifying different types of tumors than human physicians (6–8). Previous studies have investigated deep learning-based approaches to discriminate gliomas from other intracranial lesions including brain metastasis (9, 10), meningioma (10, 11), pituitary adenoma (10, 11), and acoustic neuroma (10). And other studies also reported the classification of germinoma with craniopharyngioma and pinealoblastoma (12, 13) by machine learning approaches. These reports focus on germinomas of the sellar and pineal region. Due to the uncommon incidence of germinomas of the basal ganglia, it has not been explored if routinely acquired MRI images can be utilized to differentiate germinomas from gliomas of this region. In this work, a deep learning model was established to answer this question. We enrolled 73 patients diagnosed with germinoma or glioma of the basal ganglia from two independent medical centers. **Supplementary Figure S1** shows typical T1-weighted contrast images of glioma and germinoma patients enrolled in this study, with the characteristic irregular enhancement of tumor bulk and cyst formation, which are similar between these two tumor types (14–18).

The purpose of the study is to provide aid in preoperative decision-making with a classification system for intracranial germinomas and gliomas of the basal ganglia. Enhanced T1 MRI is routinely acquired for differential diagnosis of intracranial lesions and is what’s solely needed for the neural network we developed based on ResNet-18 (19), which provides the strong capability for future clinical translation.

## Materials and Methods

### Data Collection

**Figure 1:**
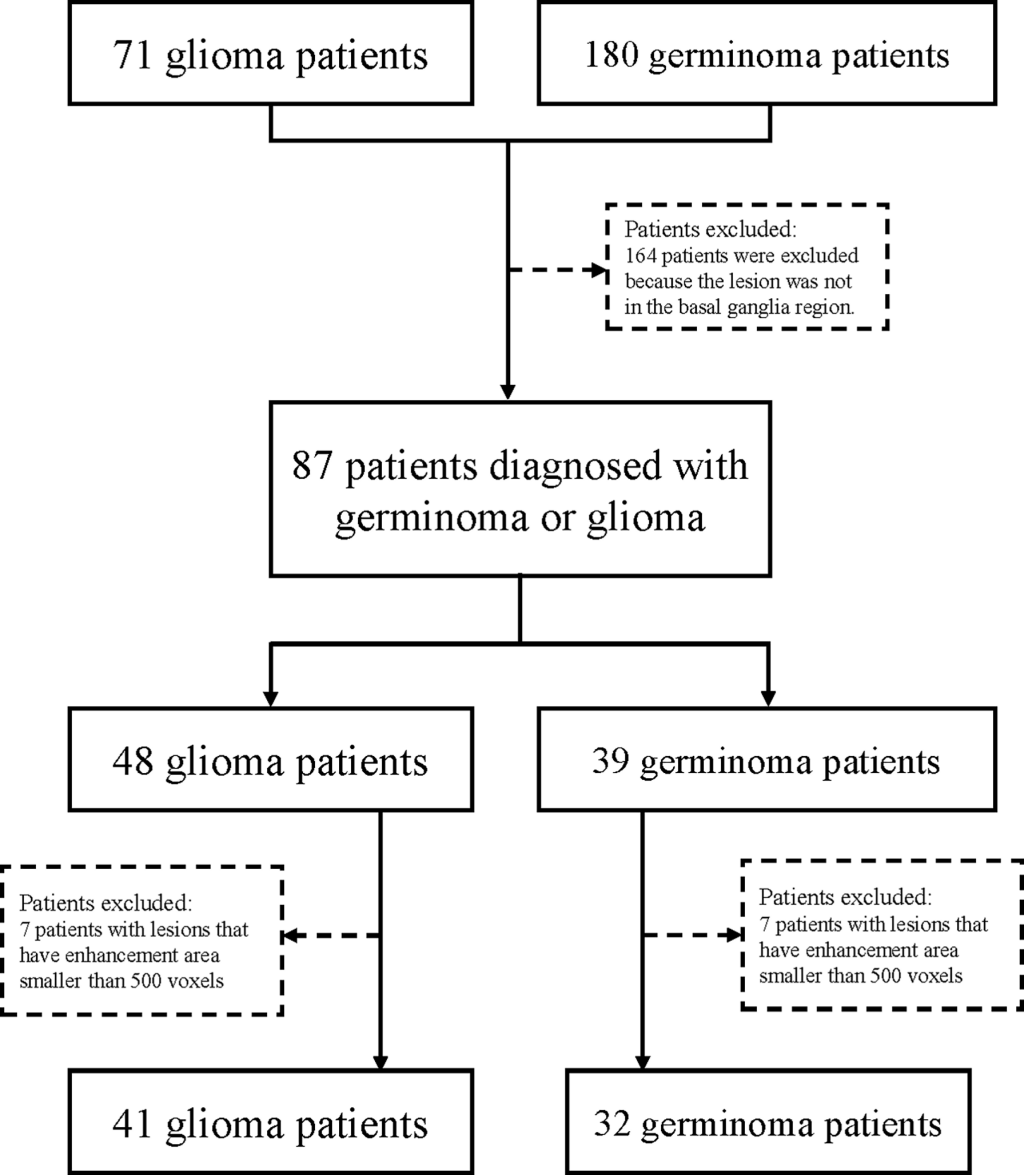
Study flowchart.

Multi-center data for a total of 180 germinomas and 71 glioma patients were retrieved from the databases of Xiangya Hospital and Sanbo Brain hospital from 2010 to 2018. Brain MRI imaging was performed as part of routine clinical care on scanners from various manufacturers with different magnetic field strengths (**Table 1**) and acquisition parameters (**Table 2**). **Supplementary Figure S2** showed the distribution of voxel geometry. A total of 39 germinoma and 48 glioma patients had lesions of the basal ganglia confirmed by immunohistochemistry (**Figure 1**). The inclusion criterion was: lesions with enhancement areas larger than 500 resliced voxels (average voxel size, 0.52 mm×0.52 mm×4.74 mm); 7 germinoma and 7 glioma patients were excluded for this reason or not having enhanced T1 image at all. There were no exclusion criteria based on age, gender, or race. Demographic and clinical data, including gender, age, and race, were retrieved from electronic medical records (**Table 3**).

**Table 1:** Clinical MRI Scanners Used.

Manufacturers and magnetic field strength	No. of patients
**All manufactures**	
Total at 1.5 T	56 (76.7%)*
Total at 3 T	17 (23.3%)
**Alltech Medical Systems**	
1.5 T	4 (5.4%)
**GE Medical Systems**	
3 T	7 (9.6%)
**Philips Medical Systems**	
1.5 T	19 (26.0%)
**SIEMENS**	
1.5 T	26 (35.6%)
3 T	10 (13.7%)
**TOSHIBA**	
1.5T	7 (9.6%)

*Numbers in parentheses are percentages.

**Table 2:** Summary of Acquisition Parameters in this study.

Parameter	Minimum	Median	Maximum
**MRI image with 3 T scanner**			
T1-weighted postcontrast MRI TE (msec)	2.37	2.98	26.82
T1-weighted postcontrast MRI TR (msec)	500	2200	2741.04
T1-weighted postcontrast MRI typical voxel size (mm)	0.72 × 0.72 × 0.9	1 × 1 × 1	1 × 1 × 5
T1-weighted postcontrast MRI typical matrix size	230 × 230	512 × 512	640 × 640
**MRI image with 1.5 T scanner**			
T1-weighted postcontrast MRI TE (msec)	4.6	10	15.7
T1-weighted postcontrast MRI TR (msec)	25	400	2100
T1-weighted postcontrast MRI typical voxel size (mm)	0.30 × 0.30 × 2	0.45 × 0.45 × 5	0.72 × 0.72 × 5
T1-weighted postcontrast MRI typical matrix size	256 × 256	512 × 416	1024 × 1024

TE, echo time; TR, repetition time.

**Table 3:** Clinical characteristics of the cohort.

Characteristics		
**Diagnosis**	Glioma	Germinoma
**No. of patients**	41	32
**Age at first diagnosis**	43 years median; 6-67 years range	13.5 years median; 7-44 years range
**Gender**		
Female	18	8
Male	23	24
**Number of lesions**		
1	37	26
>1	4	6

Therefore, a total of 73 patients (31.07 ± 18.21 years old, varying from 6 to 67 years; M: F = 47:26) were included in the final study cohort (**Table 3**). The study was approved by the institutional review board of Xiangya Hospital and Sanbo Hospital, and informed consent was waived due to the retrospective nature of this study. The study was conducted in accordance with the Declaration of Helsinki.

### Image Preprocessing and Lesion Labeling

Pre-segmentation image registration was performed with both T1-weighted contrast-enhanced (T1C) and T2-weighted fluid-attenuated inversion recovery (FLAIR) images; affine images were co-registered into the same geometric space using the Elastix toolbox (20). Image transformation and re-slicing were performed using TorchIO (21) scripts (https://github.com/fepegar/torchio); images series were resliced into an average voxel size of 0.52×0.52×4.74 mm to minimize biases in the interpolation. All the T1C and FLAIR images were used for the segmentation of enhancing tumors and peritumoral edemas, respectively. Delineation of tumor boundaries was performed in a semi-automated fashion on a slice-by-slice basis using the ITK-SNAP software, an open-source 3D image analysis kit (22). The segmented T1C and FLAIR images were reviewed for tumor delineation and consistency by two neuroradiologists (CT and NY with over 8 and 6 years of experience, respectively). The delineated images of the two segmented tumor phenotypes (enhancing tumor and peritumoral edema) were exported for further analysis. Lesions smaller than 500 voxels (about 0.65 cm^3^) were excluded for the following reasons. Small lesions like this could not be reliably segmented, typically such a small lesion will only appear on a single slice or two which makes the image less representative and reliable for feature extraction. In patients with > 1 lesion, all the lesions larger than 500 voxels were included in the analysis. The entire dataset contained a total of 93 lesions (45 germinoma lesions and 48 glioma lesions) from 73 patients (**Figure 1**).

### Data Argumentation and Transfer Learning

We adapted a ResNet18 architecture pre-trained on the ImageNet datasets (19). We only used T1C images to train the classification model.

1. Slice selection, the center slice for each lesion was selected.
2. Conversion of a grayscale image to a 3-channel image. Three strategies were compared.
  - No transformation (original gray-scale MRI images).
  - Upper and lower slices together with the center slice were stacked as R, G and B channels respectively.
  - Use the Jet color map to linearly transform the T1C gray image into an RGB image.
3. Image size normalization. Images were resized to 224 × 224.

**Figure 2:**
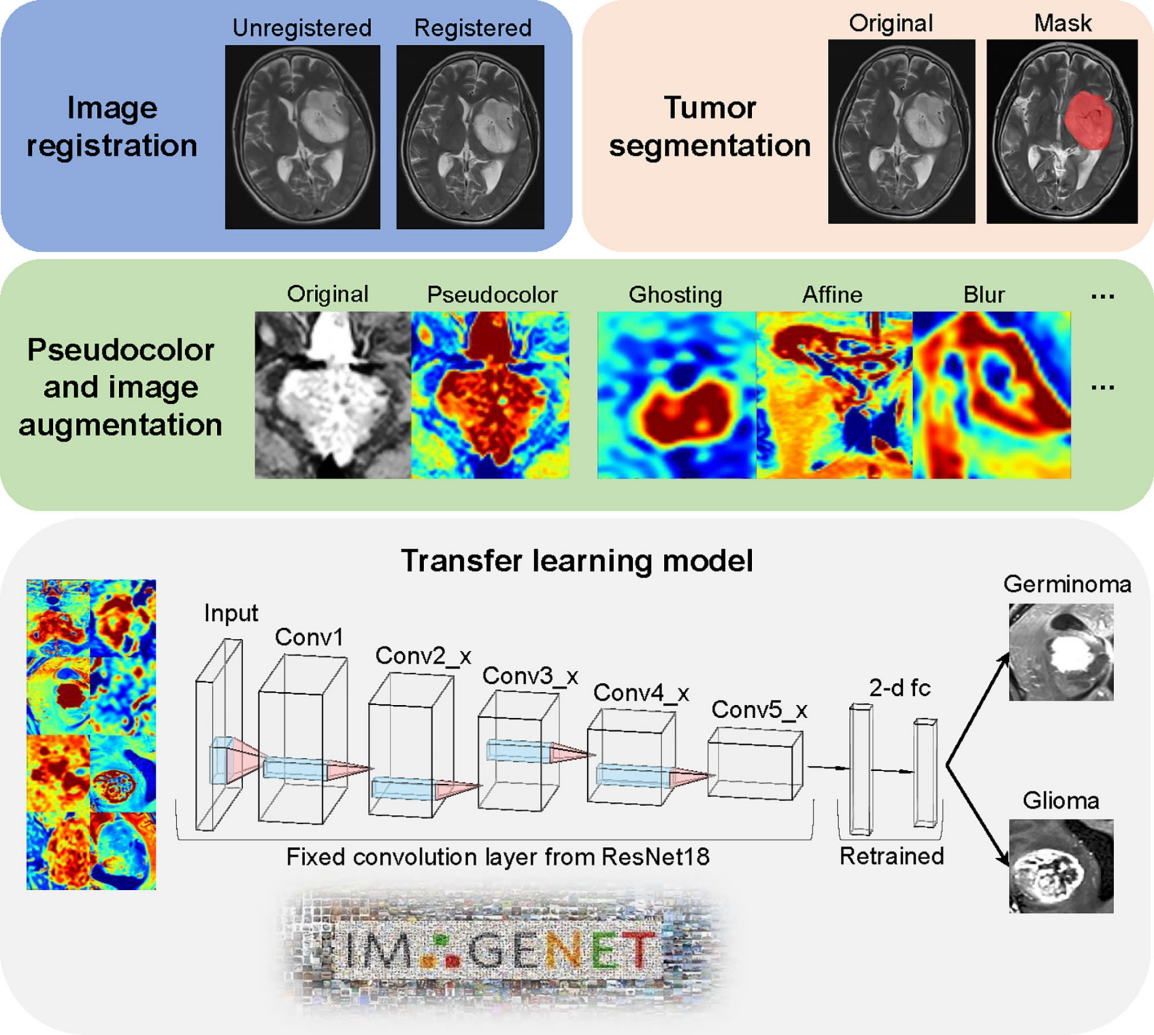
Image processing and model architecture. Image preprocessing included two major steps (image registration and tumor segmentation). The jet colormap was applied to gray-scaled MRI images, followed by the use of 6 image augmentation techniques. Convolution layers from pre-trained ResNet18 were fixed as a feature extractor. The final 2-dimension classifier was retrained to fit our data.

Due to a limited number of patients, data augmentation was performed on-the-fly to prevent overfitting. More specifically, 6 data augmentation techniques were implemented, including random flip, random affine, random blur, random ghosting, random motion, and random elastic deformation. Examples of the augmented images are shown in **Figure 2**.

The retraining process consisted of two steps, including initializing the convolutional layers with loaded pre-trained weights that trained based on the ImageNet data, and freezing all convolutional layers and fine-tuning the classification layer. By resetting the finial layer into 2 and changing the loss function into cross-entropy, our model was able to implement the pre-learned image feature extraction pattern to this tumor classification task and make prediction. To compare the model performance, we also used the same deep learning architecture to train our data from scratch. All the parameters were set identical with the pre-trained model except the randomly initial weights of convolutional layers. ResNet18 model was used from *Torchvision.models* (0.11.0).

The ResNet18 with and without pre-trained models were trained on an Ubuntu 18.04 workstation with 1 Intel Core i9-7940 CPU, using an NVIDIA GTX 1080Ti 11GB GPU, with 256 GB available system RAM. Training in all categories was run for 100 epochs or 100 steps by stochastic gradient descent in batches of 12, using an SGD Optimizer with momentum 0.9. The learning rate was set as 0.001 for all layers and utilized with a decay rate of 0.1 each 4 steps until the model gradually reached convergence. In this study, 5-fold cross-validation was performed to train the model. In each fold, 80% cases were used as the training set and the rest were used as the validation set. The training and validation were performed with *Pytorch (* https://pytorch.org/ *)* on *Python* 3.8.0.

### Model Visualization

Class activation mapping (CAM) was performed to identify the areas contributing the most to the model, as described by Zhou and collaborators (23). CAM can serve as a quietly powerful approach for the reason that they enhance image regions contributed more to the output of the model and denote the model’s confidence in the prediction. Specifically, for a unit *k* in a layer *l*, CAM calculates the importance score of *k* for class *c* and follows with visualizing the importance *via* a heatmap. We added a global average pooling layer after all the convolutional layer, which helps find all the discriminative regions. Feature maps of *l* before activation was visualized by CAM, then a heatmap was superimposed on input images.

### Statistical Analysis and Visualization

The performance statistics of the models were analyzed with the R programing language (v 4.1.2). Visualization and calculation of AUC (Area under the curve) and standard deviation of the 5-fold cross-validation was calculated with R package *precrec* (v 0.12.7) (24). In related analysis, lesion size is defined as the volumetric size of the enhanced area in T1C images. Lesions larger than median size are defined as large lesions, and lesions equivalent to or smaller than median size are defined as small lesions. Statistical tests of mean values were performed with Wilcoxon signed-rank test unless specified otherwise.

## Results

### Development of a Transfer Learning Model to Distinguish Germinomas From Gliomas

**Figure 3:**
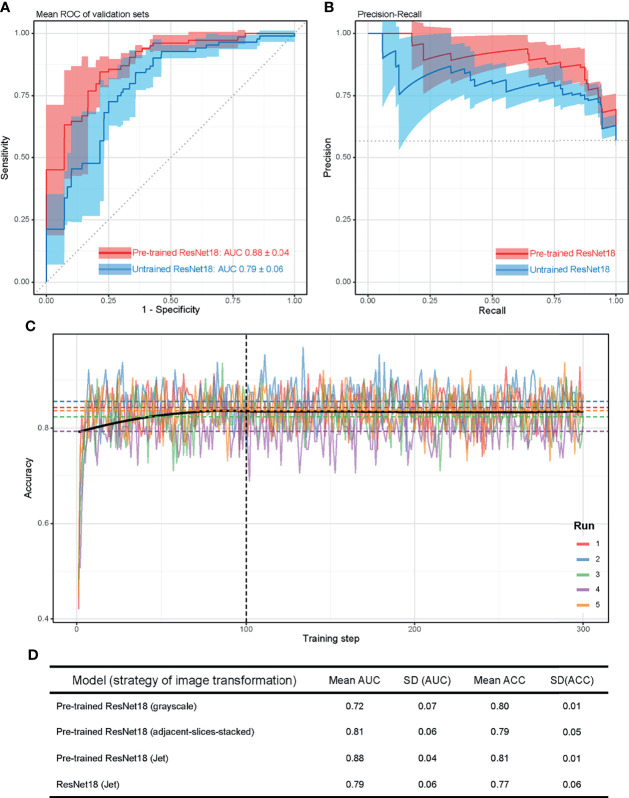
Model evaluation. **(A)** Mean ROC in validation sets for the 5 runs. AUC = 0.88 ± 0.04 (mean ± standard deviation [SD]). Red line represents transfer learning on ResNet18 pre-trained on ImageNet, blue line represents training of ResNet18 from scratch. **(B)** Precision-recall curve for the 5 runs. Red line represents transfer learning on ResNet18 pre-trained on ImageNet, blue line represents training of ResNet18 from scratch. **(C)** Accuracy of the model during training. Dotted lines indicate mean accuracy from training steps 100 to 300, of the five-fold training. Black line is the loess fitting of accuracy of n-fold cross-validation at each training step. **(D)** Mean AUC and ACC of the four model we trained in this study. SD, standard deviation. ACC, accuracy.

As described in the method, 3 strategies of image transformation were compared. First, we trained the model with the original gray-scale MRI image, the model reached AUCs of 0.72 ± 0.07 (mean ± standard deviation [SD]) (**Supplementary Figure 3A**). Second, the model trained on adjacent-slices-stacked images reached AUCs of 0.81 ± 0.06 (mean ± SD) (Sup. Figure 3A). Third, the model trained with the Jet colormap-transformed images reached AUCs of 0.88 ± 0.04 (mean ± SD) (**Figure 3A**). Image transformation with the Jet colormap seems to be the best strategy for our dataset based on the ROC and the precision-recall curve (**Figures 3A, B, D** and **Supplementary Figures 3A, B**). The best model reached accuracy levels of 0.81 ± 0.01 in the validation set (**Figures 3C, D**). The precision-recall curves of these models indicate that when more information is provided to the model, the result is slightly better.

To test the effectiveness of transfer learning and provide a performance benchmark, we also trained ResNet18 on Jet colormap-transformed images from scratch. Unsurprisingly, the performance is worse than the pre-trained one, reaching AUCs of 0.79 ± 0.06 (mean ± SD), as shown in **Figures 3A, B** (The blue lines).

The mean lesion size of germinoma is smaller than glioma **Supplementary Figures 4A, B**. To test whether lesion size affects model performance, we performed chi-square (χ^2^) test on contingency tables of True-False prediction and tumor size of the 5-fold cross-validation **Supplementary Figures 4C, D**. No significant difference in the distributions of predicting correctness between small and larger lesions is found. This proved the model’s ability to generalize over various tumor sizes. Still, the median lesion size of correct prediction is slightly larger than incorrect prediction. Indicate that the model might perform better with larger lesions (**Supplementary Figures 4C, D**). Additionally, the model was tested with lesions excluded in the original analysis (smaller than 500 voxels), reached AUCs of 0.64 ± 0.07 (mean ± SD), this indicates that our model might not be suitable for ultra-small lesions (**Supplementary Figures 3C, D**).

### The Class Activation Map Reveals a Location Preference That the Model Focuses on for Different Tumors

The neural network-based machine learning model was more sophisticated and less interpretable than traditional methods. Therefore, we applied CAM (23) to probe the model after training. Pixels on a class activation map represent the superimposed activation strength of each unit in the last convolutional layer, which can be used to detect the regions on which the model mostly focuses; in other words, this could reveal the location preference that the model focuses on for different tumors.

**Figure 4:**
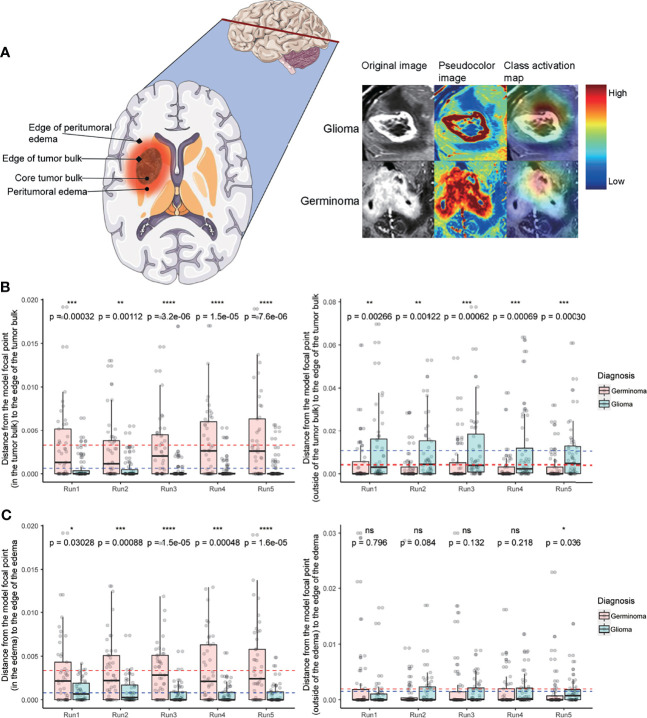
Class activation map analysis. **(A)** A schematic of a tumor’s physical structure (left), a schematic of the image with pseudo-color (middle), and a superimposed class activation map (right). On the class activation map that is overlaid on the original image, the color bar indicates weights (red and blue for high and low, respectively). **(B)** Distance from the model’s focal point (in the tumor bulk) to the edge of the tumor bulk (left). Distance from the model’s focal point (outside of the tumor bulk) to the edge of the tumor bulk (right). **(C)** Distance from the model’s focal point (in the edema) to the edge of the edema (left). Distance from the model’s focal point (outside of the edema) to the edge of the edema (right). In C and D, dotted lines indicate the mean distance (red and blue for germinoma and glioma, respectively). Distances are normalized by tumor size. Wilcoxon signed-rank test, *P < 0.05, **P < 0.01, ***P < 0.001, ****P < 0.0001. ns, not significant.

Both germinoma and glioma are characterized by a bulk region (or core region) and a peritumoral edema region (**Figure 4A**, left). To examine if the model had “attention” preferences for different regions, we overlaid the class activation map onto the original image (**Figure 4A**, right). Whether the focal points were located in or outside of the tumor bulk, they were significantly more likely to be centric for germinoma than glioma cases in all five cross-validation runs (**Figure 4B**). When the focal points were located in the edema, they were significantly more likely to stay away from the outer edge of the edema for germinoma than glioma cases (**Figure 4C**). No significant differences in the distance were found from the focal points (when outside of the edema) to the outer edge of the edema (**Figure 4C**), from the focal points to the center mass of the tumor bulk, and from the focal points to the center mass of the peritumoral edema **Supplementary Figure 5** between these two tumor types. In other words, there was a tendency for the model to focus on the peritumoral edema region of gliomas and the tumor bulk for germinomas. These findings indicated different properties of these two types of tumors in terms of physical structure, which could help discriminate them.

## Discussions

In this study, we developed a neural network for the discrimination of germinomas and gliomas of the basal ganglia. Most previous reports of germinoma are case reports or prognosis analyses based on tens of cases. To our best knowledge, this is the largest cohort of basal ganglia germinoma and the first quantitative MRI image analysis. The rarity of this disease with the specific location makes it difficult to collect enough data to train a convolutional neural network from scratch. In this case, we applied two commonly implemented techniques. The first of which is data augmentation to increase the dataset size and fight overfitting. The second is transfer learning to dramatically reduce the number of parameters to fit (25, 26).

It is reported that color information improves the performance on object recognition tasks of human participants (27). As color image encodes more visual information than grayscale image. It is less explored if colorized medical image improves accuracy in diagnostic imaging when examined by a human radiologist. Kather, J. N. et al. showed that radiologists can detect cancer tissue on colorized MRI images, with equivalent performance on grayscale images, while receiving almost no extra previous training (28). Our transfer learning method is based on the ResNet18, pre-trained on the ImageNet dataset, a natural scene database consisting of over 14 million manually annotated RGB color images of over 1000 categories. The above inspired us to test if deep learning models also have better performance with colorized medical images in classification tasks. Currently, there is no well-acknowledged method for adding pseudo color to medical images. Previously reported method includes linear color conversion from grayscale to a color map (29), triplicate the grayscale channel to synthesize color image (30, 31), concatenating three independent slices from one or cross different series (planes) (32–35). In this study, we thoroughly benchmarked these methods. The results showed that linear transformation to a color map yielded the highest AUC in our dataset. This provides a valuable reference for future implementation of neural networks on medical imaging.

Neural network-based machine learning models are infamously known as “black boxes”. Therefore, CAM was implemented to improve interpretability, and to shed light on the decision-making process of the network, thus ensuring the focal point of the model doesn’t fall in irrelevant areas. A few misclassified cases were shown in **Supplementary Figure 6**. For misclassified germinomas **Supplementary Figure 6A**, focal points of the model are located near ventricle structures and irrelevant peritumoral white matter. For misclassified gliomas **Supplementary Figure 6B**, focal points of the model located at the tumor bulk or edge of it. Previous reports showed that germinomas of the basal ganglia is characterized with minimal peritumoral edema (36, 37). As for glioma, peritumoral edema is a classic feature of MRI T2 image especially for high-grade glioma (38, 39). This might explain why the model fails on these specific cases and why it emphasizes peritumoral edema of gliomas. The model attention areas provide a valuable reference for physicians in case of suspected germinomas of the basal ganglia to avoid misdiagnosis. We delineated the tumor regions manually for CAM-related analysis, integrating automatic classification algorithms and auto segmentation of tumor will be explored in the future for the deployment of such models in clinical practice.

Medical images are volumetric datasets as they typically contain slices of body segments and organs. In this study, we developed a neural network model based on a single slice of T1C MRI image, which is routinely acquired in medical practices. Unlike the traditional radiomics based model, no laborious slice-by-slice segmentation and labeling is required. The moderate AUC of the model provides a stronger capability of clinical translation. The increased number of parameters and the higher computational complexity limited the application of 3D CNN currently, but it might perform better in a larger dataset than single-slice-based CNN.

This study had several limitations. First, the jet colormap was still not representative and interpretable for different regions on the image, such as the tumor bulk, edema, and normal tissues. In future research, a customized colormap both suitable for machine learning algorithms and human interpretation of MRI gray-scaled images should be developed. Second, although we implemented a good visualization method to identify the focus of this model and discovered the differences for both tumors, the biological meanings of the features of the model attention mechanism still need to be explored further. Interpretable CNN, which trains the kernel to represent the specific meaning of parts on objects, could integrate biological knowledge for better interpretability (40), which should be implemented in the future. Third, the dataset was still small even though we gathered data from two large medical centers. Data augmentation was used to alleviate this problem, still, the repeatability and robustness of the model should be validated with external data, if possible. Finally, our model showed moderate ability to generalize over various tumor sizes, but the model might have better performance on large lesions.

## Conclusions

A transfer learning classifier for germinomas and gliomas of the basal ganglia was built, reaching a mean AUC of 0.88 and a mean accuracy of 0.81 in the validation set. By employing class activation mapping, we found the model was focused on the peritumoral edema region of gliomas and the tumor bulk for germinomas.

## Data Availability Statement

The original contributions presented in the study are included in the article/**Supplementary Material**. Further inquiries can be directed to the corresponding authors.

## Ethics Statement

The studies involving human participants were reviewed and approved by the ethics committee of Xiangya Hospital and the ethics committee of Sanbo Hospital. Written informed consent from the participants’ legal guardian/next of kin was not required to participate in this study in accordance with the national legislation and the institutional requirements.

## Author Contributions

XLi, SL designed this study, NY, QY, ZC performed the analysis, NY, QY wrote the manuscript, NY, CT finished the segmentation of all MRI images, PL, XLin, XLiu, YX contributed in acquisition of the data used in this study. All authors read and approved the final manuscript.

## Funding

This work was supported by the National Natural Science Foundation of China (for XLi, Grant No. 81770781 and No. 81472594) and Beijing Natural Science Foundation (for SL, grant No. 7182076 and H2018201306).

## Conflict of Interest

The authors declare that the research was conducted in the absence of any commercial or financial relationships that could be construed as a potential conflict of interest.

## Publisher’s Note

All claims expressed in this article are solely those of the authors and do not necessarily represent those of their affiliated organizations, or those of the publisher, the editors and the reviewers. Any product that may be evaluated in this article, or claim that may be made by its manufacturer, is not guaranteed or endorsed by the publisher.
