# Supplementary material for: Classification of Gliomas and Germinomas of the Basal Ganglia by Transfer Learning
Source: Front Oncol. 2022 Mar 3;12:844197. doi: 10.3389/fonc.2022.844197 (PMC8928458; doi:10.3389/fonc.2022.844197)
Supplement: Supplementary file 1 [file Image_1.pdf]

Supplementary Material

1 Supplementary Figures

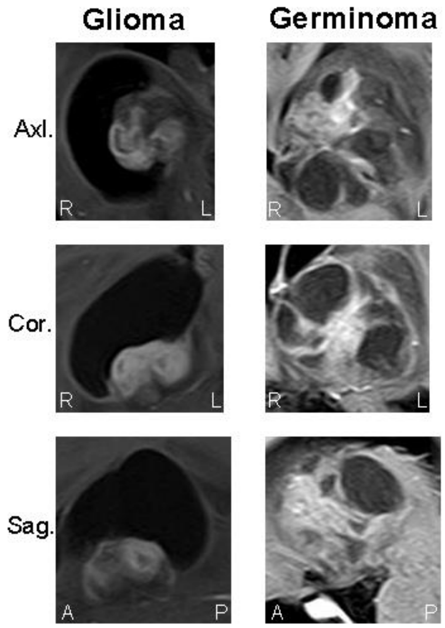

Supplementary Figure 1. Typical T1-weighted contrast MRI image of glioma patient (left) and germinoma patient (right).

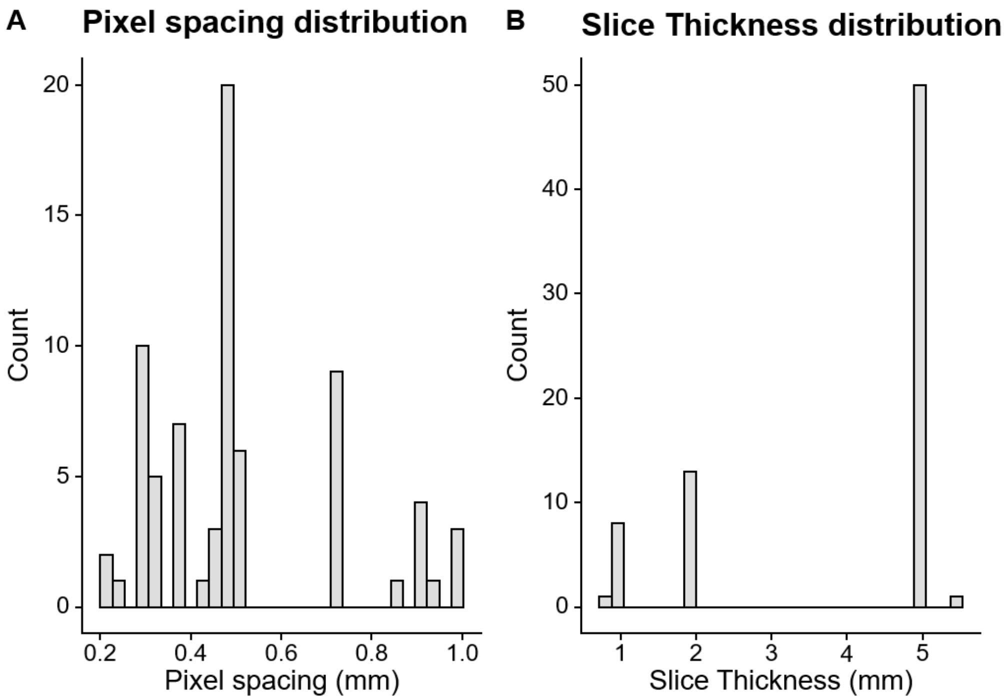

Supplementary Figure 2. distribution of voxel geometry.

A, voxel size is described as $x \times y \times z$ mm, pixel spacing is the length of *x* and *y*.

B, slice thickness of axial image which is the the length of aforementioned *z*.

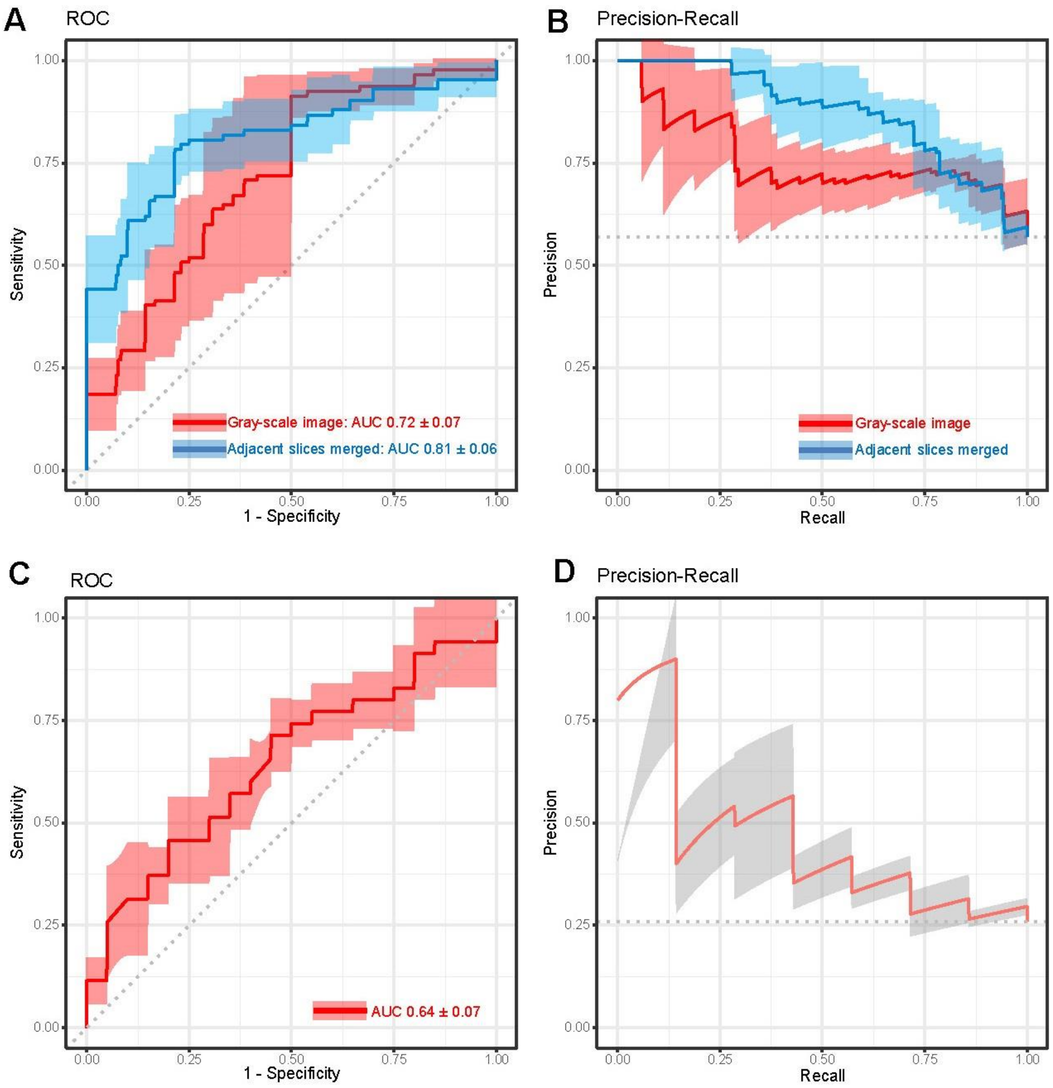

Supplementary Figure 3. Model performance of different channel transformation strategies and ultra-small lesions.

A, Mean ROC in validation sets for the 5 runs. Red line represents transfer learning with original gray-scale MRI images, blue line represents transfer learning with pseudo-color images generate by merging adjacent slices.

B, Precision-recall curve for the 5 runs. Red line represents transfer learning with original gray-scale MRI images, blue line represents transfer learning with pseudo-color images generate by merging adjacent slices.

C, Mean ROC in validation sets for the 5 runs. Pre-trained ResNet18 tested on ultra-small lesions (smaller than 500 voxels) with Jet colormap-transformed images.

D, Precision-recall curve for the 5 runs. Pre-trained ResNet18 tested on ultra-small lesions (smaller than 500 voxels) with Jet colormap-transformed images.

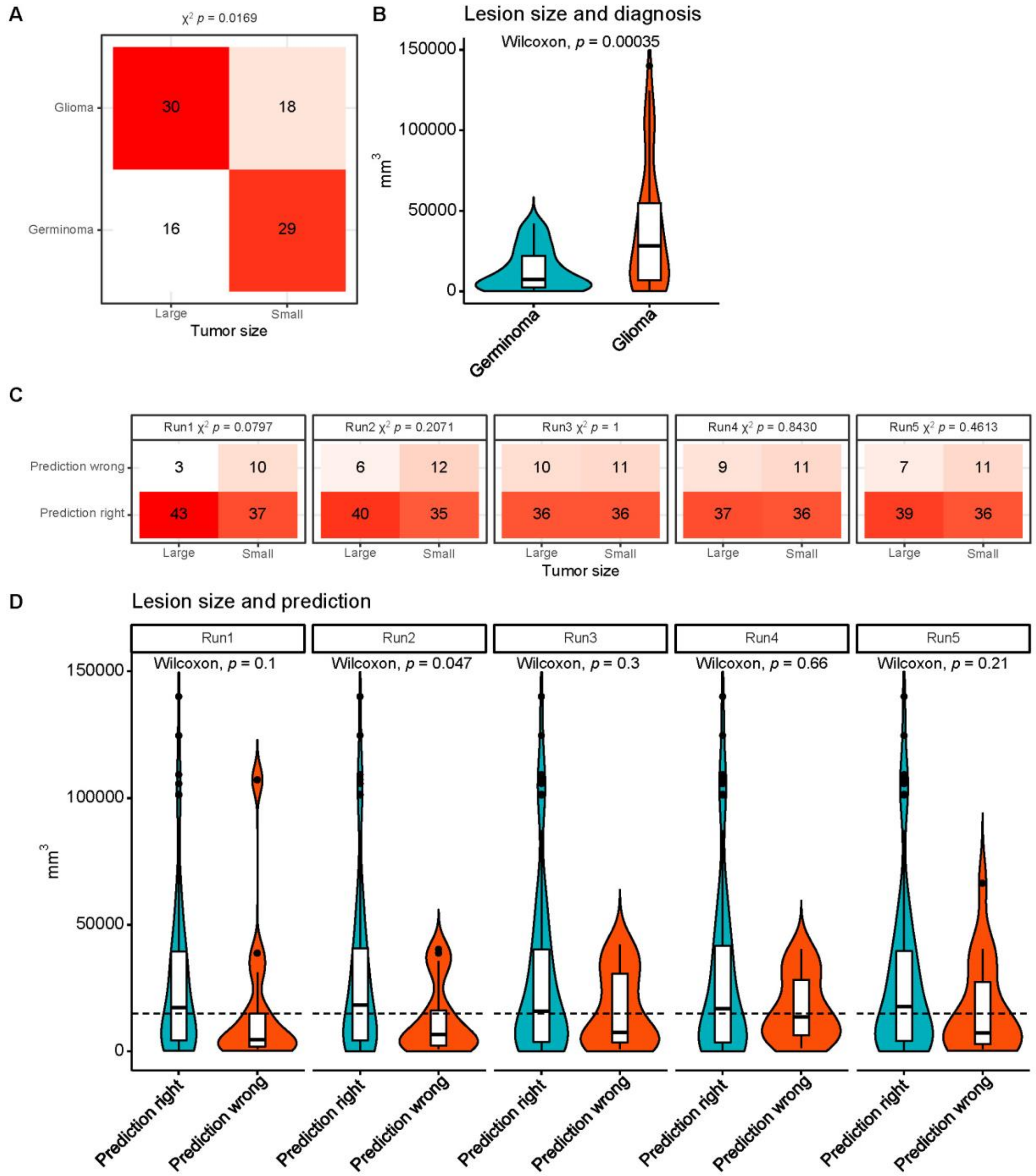

Supplementary Figure 4. Lesion size and the performance of the model.

A, contingency table of tumor size and diagnosis. *P* value is calculated with chi-square (χ^2) statistic.

B, lesion size distribution of germinomas and gliomas, mean size tested with Wilcoxon signed-rank test. Size of germinomas ranges from 654.3 to 42134.6 mm^3, mean size is 13355.2 ± 12999.0 mm^3

(mean ±SD). Size of gliomas ranges from 935.0 to 140065.9 mm^3, average size is 38357.3 ± 37946.9 mm^3 (mean ± SD).

C, contingency tables of tumor size and model prediction (of test set) of the 5-fold cross-validation. Tumor size split into large and small group by median size. *P* values were calculated with chi-square (χ^2) statistic.

D, lesion size distribution of correct or incorrect prediction of the model, mean size tested with Wilcoxon signed-rank test. Dotted line denote the median size.

Lesion size is defined as the volumetric size of the enhanced area in T1C images. Lesions larger than median size are defined as large lesions, and lesions equivalent to or smaller than median size are defined as small lesions.

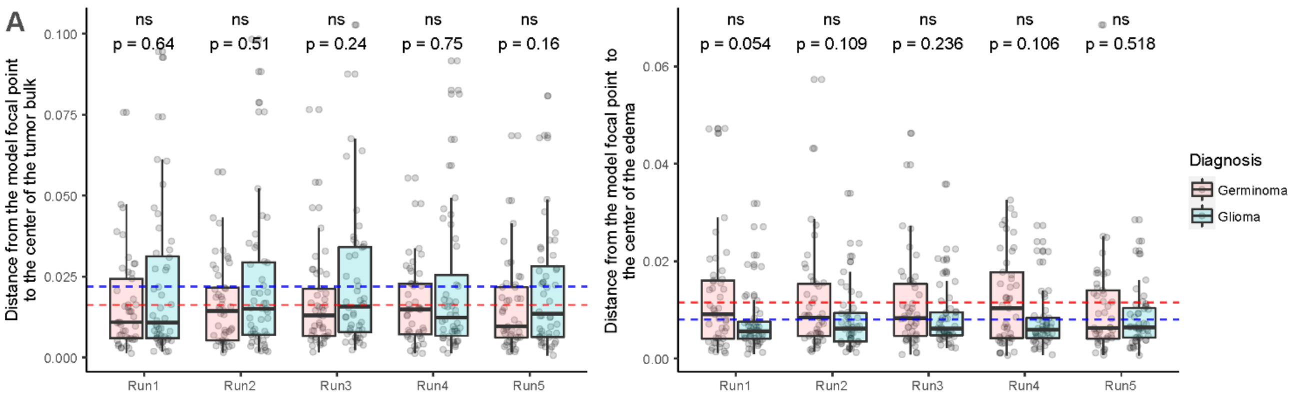

Supplementary Figure 5. Class activation maps analysis.

A, Distance from the model focal point to the center of the tumor (left), distance from the model focal point to the center of the edema (right).

Wilcoxon signed-rank test, ns *P* > 0.05

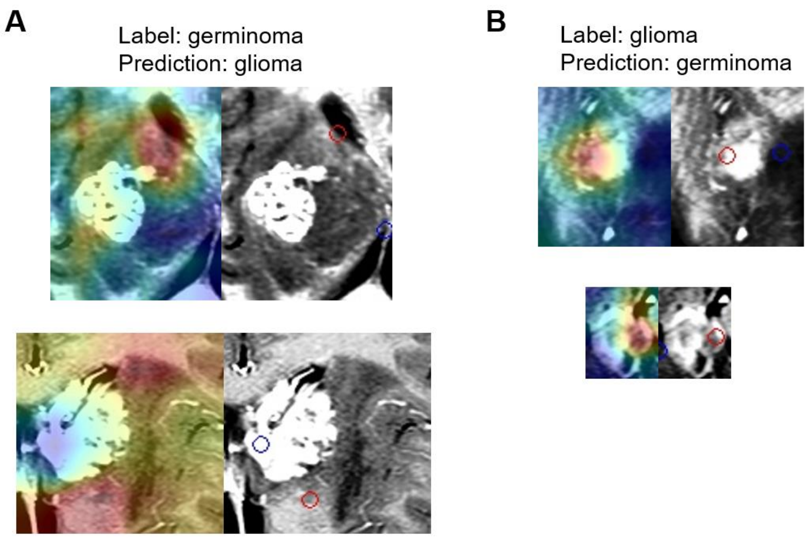

Supplementary Figure 6. Misclassified cases.

A, misclassified germinoma.

B, misclassified glioma.

In the image with pseudo-color, red denotes more “attention”, blue denotes less. Circle on gray-scale image indicate location of highest (Red circle) or lowest (Blue circle) “attention”.
